# Portfolio Optimization Model for Gold and Bitcoin Based on Weighted Unidirectional Dual-Layer LSTM Model and SMA-Slope Strategy

**DOI:** 10.1155/2022/1869897

**Published:** 2022-06-08

**Authors:** Qianyi Xue, Yuewei Ling, Bingwei Tian

**Affiliations:** ^1^Institute for Disaster Management and Reconstruction, Sichuan University, Chengdu 610207, China; ^2^College of Computer Science, Sichuan University, Chengdu 610207, China; ^3^Pittsburgh Institute, Sichuan University, Chengdu 610207, China; ^4^West China Hospital of Sichuan University, Chengdu 610041, China

## Abstract

Portfolio optimization is one of the most complex problems in the financial field, and technical analysis is a popular tool to find an optimal solution that maximizes the yields. This paper establishes a portfolio optimization model consisting of a weighted unidirectional dual-layer LSTM model and an SMA-slope strategy. The weighted unidirectional dual-layer LSTM model is developed to predict the daily prices of gold/Bitcoin, which addresses the traditional problem of prediction lag. Based on the predicted prices and comparison of two representative investment strategies, simple moving average (SMA) and Bollinger bands (BB), this paper adopts a new investment strategy, SMA-slope strategy, which introduces the concept of *k*-slope to measure the daily ups and downs of gold/Bitcoin. As two typical financial products, gold and Bitcoin are opposite in terms of their characteristics, which may represent many existing financial products in investors' portfolios. With a principle of $1000, this paper conducts a five-year simulation of gold and Bitcoin trading from 11 September 2016 to 10 September 2021. To compensate for the SMA and BB that may miss buying and selling points, 4 different parameters' values in the *k*-slope are obtained through particle swarm optimization simulation. Also, the simulation results imply that the proposed portfolio optimization model contributes to helping investors make investment decisions with high profitability.

## 1. Introduction

Industry 4.0 was first introduced in 2013, defining “the transition from a time when people worked with computers to when computers work without humans.” The world is witnessing the development of information technology and the widespread use of computers. The emergence of Industry 4.0 has impacted global financial markets and continues to drive technological iterations in the financial field [1]. Portfolio optimization has always been a popular topic in modern financial research, and investors with different capital levels have to face the problem of portfolio selection [2]. The optimal portfolio selection yields the highest expected return within an acceptable risk range [3], but high returns usually come with high risks [4]. Technical analysis uses historical long-term and short-term stock trends to help investors make informed and profitable trading decisions [5]. Zhu and Zhou pointed out that technical analysis can add value to stocks when their returns are predictable. It contributes to identifying trading opportunities when there are uncertainties about stock returns [6]. Portfolio optimization in the real world is a very difficult and complex mathematical problem [7]. Based on the technical analysis, the portfolio optimization of gold and Bitcoin is divided into two subproblems, daily price prediction and decision algorithms of investment strategies.

### 1.1. Price Prediction

Intelligence in manufacturing is considered an essential hallmark of Industry 4.0, driven by the boom and maturity of new information and communication technologies applied to industrial processes and products [8]. The growth of available information in industrial plants has contributed to the widespread use of machine learning in addressing specific industrial needs [9]. In the era of Industry 4.0, prediction is a hot topic, especially the ability to predict events related to industrial assets and production processes [10]. With the vigorous development of artificial intelligence (AI), many optimization techniques based on machine learning and deep learning have favored many investors, which are applied to predict the prices of financial products, especially stock price prediction. There are many optimal methods for stock price prediction, but no perfect solution has been developed yet. Stock prices are affected by multiple factors in the stock market, and the mechanisms of these factors are incredibly complex. Changes in investor sentiment are also a major cause for changes in stock prices, which are usually analyzed by sentiment analysis. With evaluation of the causal relationship between VIX and BTC, Chi concluded that Bitcoin is not a safe asset in a climate of fear, which contributes to obtaining the most risk returns [11]. Moreover, some external factors like the black swan event are difficult to predict but significantly impact stock prices. For example, the COVID-19 pandemic is a black swan event for financial markets [12].

Time-series forecasting is traditionally performed in econometrics using the autoregressive integrated moving average (ARIMA) model [13]. But some problems of the ARIMA model are gradually emerging as it is applied in different fields: (1) As a linear model, it is difficult for the ARIMA model to establish nonlinear relationships between variables. (2) Given that stock prices are usually noisy, volatile, and nonparametric, it should be a complex nonlinear problem. However, the error of the ARIMA model cannot have a constant standard deviation. Kane found that although the problem in the ARIMA model can be solved to a certain extent using the ARIMA-GARCH model, there are some problems of the optimization of parameters in the generalized autoregressive conditional heteroskedasticity (GARCH) model [14]. Sai optimized the kernel function of the support vector machine (SVM) and used the optimized model to predict and analyze the investment stock index, which performed significantly better than the ARIMA model [15]. With the development of neural networks and the superiority of long short-term memory (LSTM) in natural language processing tasks, LSTM has been applied to the same time-series stock price prediction problem. Ma compared the performances of three models in stock price prediction, the ARIMA model, the artificial neural network (ANN) model, and the LSTM model. Mahas found that the LSTM model performs better because of its improvement on the vanishing gradient problem [16]. Yurtsever noticed that LSTM performed the best by comparing three multivariate time-series models (LSTM, Bi-LSTM, and GRU), using six indicators of crude oil price, consumer price index, stock market index, effective exchange rate, interest rate, and gold price as model inputs [17]. Saifi proved that the LSTM-based prediction model is slightly better than other prediction models (GRU, DNN, and RNN) in Bitcoin price prediction (regression) [18]. Selvin et al. tested the performance of CNN, LSTM, and RNN on the same sliding window and concluded that CNN can capture short-term trend changes and achieve better results than LSTM and RNN because CNN does not rely on any previous information to make predictions. It only uses the current data window to make predictions [19]. Fleischer et al. used LSTM to evaluate predictions for cryptocurrencies such as Bitcoin and pointed out that the results seemed promising as the predicted values deviate very little from the true values. Still, upon closer inspection, it turned out that the prediction lags by a day since stock prices follow the random walk theory, which means that the nature of their movement follows a random walk; that is, changes in prices are not necessarily the result of previous changes [20].

### 1.2. Investment Strategy

Decision-making, a kind of human behavior aimed at achieving a specific goal, occurs in every activity of human society [21]. An investment strategy is a set of rules to guide investors in trading decisions. The right investment strategy is critical to an investor's success, which requires every investor to analyze as much of the available data as possible [22]. The simple moving average (SMA) and Bollinger bands (BB) are common investment strategies. SMA uses two moving averages, a long period and a short period moving average, which is straightforward to help investors make decisions [23]. Liu and Malik proposed a neural network-based framework to improve profit generation, where SMA effectively measures the volatility of stocks [24]. SMA has good stability, which is not influenced by temporary price fluctuations. BB consist of three lines, the upper, the middle, and the lower. BB can be used to recognize volatility and trends of the price, which allow investors to realize the breakouts. BB were applied to identify stocks with the highest profitability [25]. However, SMAs do not react promptly enough to rapid price changes at market reversal points, and BB overly reply to current market movements. The flaws of SMA and BB may cause investors to miss suitable and favorable buying and selling opportunities. In addition to providing the right time to buy and sell, the buy and sell ratio is also the focus of the investment strategy. Particle swarm optimization (PSO) was adopted to optimize the trading setups of Bitcoin [26]. Zhu et al. applied PSO to a metaheuristic approach to solving the intractability of portfolios [27]. Also, Butler and Kazakov pointed out that PSO can offer better trading results [28].

### 1.3. Contribution

This paper adopts the technical analysis to establish a portfolio optimization model based on the unidirectional dual-layer LSTM model and the SMA-slope strategy. The unidirectional dual-layer LSTM model is developed to predict gold and Bitcoin's daily average price data, which realizes one of the most important goals of Industry 4.0, intelligent prediction. It is a common approach to designing portfolios with an investment horizon greater than one year based on daily data [29]. Also, we propose a weighting method to address the traditional problem of prediction lags. The unidirectional dual-layer LSTM model is trained and tested on the two datasets, daily gold prices (London Bullion Market Association, 9 November 2021) and daily Bitcoin prices (NASDAQ, 9 November 2021). Gold and Bitcoin are two diametrically opposed financial products due to their widely varying volatility trends and opposed characteristics, such as linearity and stability. Also, this paper introduces the SMA-slope strategy. The SMA-slope strategy uses PSO to determine the optimal buying and selling ratio. It also increases the buying and selling points using the concept of *k*-slope based on the SMA strategy, which solves the insensitivity of the SMA strategy to short-term price fluctuations. The introduction of the *k*-slope reflects a kind of human behavior, as investors do not tend to change their existing views until they are convinced of new plausible trends [30]. Simulation is a key technology in the era of Industry 4.0 [31]. With an initial principal of $1,000 and different trading commissions as a prerequisite, we compare the SMA-slope strategy to the SMA and BB strategies by simulating 5 years of actual trading from 2016 to 2021.

At present, most researches focus on the price prediction and investment strategies of stocks. Based on the technical analysis of stocks, this paper considers the portfolio optimization for gold and Bitcoin. Gold and Bitcoin are typical products of the financial market, which have opposite characteristics. The study of the portfolio optimization of these two financial products contributes to optimizing the portfolio of various products with different characteristics in the financial market. Also, more attention has been paid to regression evaluation indicators like RMSE and MAPE while predicting prices. Few studies have focused on the performance of times-series models on stocks and gold/Bitcoin in terms of prediction lags. The prediction lag is a traditional problem of time-series problem, and researches have shown that LSTM can solve it to some extent [32]. This paper focuses on the prediction lag and employs a lag metric (up and down accuracy) to assess the performance. A small sliding window is used to forecast, and predicted prices for the next few days obtained by the model are weighted. The specific weights are obtained from various tests, which improve the accuracy of the rise and fall. Moreover, few papers provide a comparative analysis of the SMA strategy and Bollinger band strategy, and the buying and selling points due to these two strategy measures are not addressed. In this paper, the SMA-slope strategy is proposed based on the concept of *k*-slope, which conduces improving the sensitivity to reasonable buying and selling points.

## 2. Methodology

### 2.1. Feature Selection

Based on the literature review [33, 34] and data availability, we select 18 features: simple moving average (SMA), relative change (RC), exponential moving average (EMA), moving average convergence/divergence (MACD), relative strength index (RSI), Bollinger bands, and so on. These features will be used as input vectors to the LSTM model for training. The LSTM model is used to predict the daily average prices of gold/Bitcoin.

The simple moving average (SMA) over the last *k* days is calculated by (1) as follows:

(1) $$\begin{array}{c} {\text{SMA}}_{k}=\frac{p_{n-k+1}+p_{n-k+2}+\cdots +p_{n}}{k}=\frac{1}{k}\sum_{i=n-k+1}^{n}p_{i}, \end{array}$$

where *p*_*i*_ is the value of the gold/Bitcoin on the *i*-th day.

The relative change (RC) of the simple moving average is calculated by (2) as follows:

(2) $$\begin{array}{c} \text{RC}=\ln\left(\frac{{\text{SMA}}_{1}}{{\text{SMA}}_{5}}\right), \end{array}$$

where SMA_1_ is the simple moving average over the last 1 day and SMA_5_ is the simple moving average over the last 5 days.

The exponential moving average (EMA) over the last *n* days is calculated by (3) as follows:

(3) $$\begin{array}{c} {\text{EMA}}_{i}=\left\{\begin{array}{cc} p_{1}, & i=1,\\ \left(\frac{n-1}{n+1}\right){\text{EMA}}_{i-1}+\left(\frac{2}{n+1}\right)p_{i}, & i>1, \end{array}\right) \end{array}$$

where *p*_*i*_ is the value of the gold/Bitcoin on the *i*-th day.

The differential value (DIF) is calculated by (4) as follows:

(4) $$\begin{array}{c} {\text{DIF}}_{i}={\text{EMA}}_{i}\left(12\right)-{\text{EMA}}_{i}\left(26\right), \end{array}$$

where EMA_*i*_(12) is the exponential moving average over the last 12 days on the *i*-th day and EMA_*i*_(26) is the exponential moving average over the last 26 days on the *i*-th day.

The differential exponential average (DEA) is calculated by (5) as follows:

(5) $$\begin{array}{c} {\text{DEA}}_{i}=\left\{\begin{array}{cc} 0, & i=1,\\ \left(0.8\right){\text{DEA}}_{i-1}+\left(0.2\right){\text{DIF}}_{i}, & i>1, \end{array}\right) \end{array}$$

where DIF_*i*_ is the differential value of the gold/Bitcoin on the *i*-th day.

The moving average convergence/divergence (MACD) is calculated by (6) as follows:

(6) $$\begin{array}{c} {\text{MACD}}_{i}=2\left({\text{DIF}}_{i}-{\text{DEA}}_{i}\right), \end{array}$$

where DIF_*i*_ is the differential value of the gold/Bitcoin on the *i*-th day and DEA_*i*_ is the differential exponential average of the gold/Bitcoin on the *i*-th day.

The growth periods over the last 14 days are characterized by the value of the gold/Bitcoin being higher than the value of the previous day; that is, *p*_*i*_ > *p*_*i*−1_. The gross growth (GG) over the last 14 days is calculated by (7) as follows:

(7) $$\begin{array}{c} \text{GG}=\sum_{i=n-13}^{14}\left(p_{i}-p_{i-1}\right), \end{array}$$

where *p*_*i*_ is the value of the gold/Bitcoin on the *i*-th day.

The decline periods over the last 14 days are characterized by the value of the gold/Bitcoin being not higher than the value of the previous day; that is, *p*_*i*_ ≤ *p*_*i*−1_. The gross decline (GD) over the last 14 days is calculated by (8) as follows:

(8) $$\begin{array}{c} \text{GD}=\sum_{i=n-13}^{14}\left(p_{i-1}-p_{i}\right). \end{array}$$

The relative strength (RS) is calculated by (9) as follows:

(9) $$\begin{array}{c} \text{RS}=\frac{\text{GG}}{\text{GD}}\text{,} \end{array}$$

where GS is the gross growth and GD is the gross decline.

The relative strength index (RSI) is calculated by (10) as follows:

(10) $$\begin{array}{c} \text{RSI}=100-\frac{100}{1+\text{RS}}, \end{array}$$

where RS is the relative strength.

The Bollinger bands refer to the upper Bollinger band, middle Bollinger band, and lower Bollinger band, which can reflect the value volatility of gold/Bitcoin over time. The middle Bollinger band (MBB) over the last 20 days is calculated by (11) as follows:

(11) $$\begin{array}{c} \text{MBB}=\frac{1}{20}\sum_{i=n-19}^{n}p_{i}, \end{array}$$

where *p*_*i*_ is the value of the gold/Bitcoin on the *i*-th day.

The upper Bollinger band (UBB) over the last 20 days is calculated by (12) as follows:

(12) $$\begin{array}{c} \text{UBB}=\text{MBB}+2\sigma , \end{array}$$

where MBB is the middle Bollinger band and *σ* is the standard deviation of the value of the gold/Bitcoin over the last 20 days.

The lower Bollinger band (LBB) over the last 20 days is calculated by (13) as follows:

(13) $$\begin{array}{c} \text{LBB}=\text{MBB}-2\sigma , \end{array}$$

where MBB is the middle Bollinger band and *σ* is the standard deviation of the value of the gold/Bitcoin over the last 20 days.

### 2.2. Price Prediction with LSTM Model

**Figure 1:**
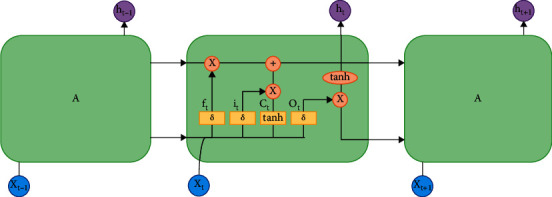
LSTM network structure.

Recurrent neural network (RNN) can reflect the sequence-related characteristics of financial time-series data, but it has the problem of gradient disappearance or gradient explosion. Also, its mining of historical information for financial time-series data is very limited. LSTM is a special RNN that can well handle the long-term dependencies of time-series data [35]. Therefore, the LSTM model is an improved RNN model, to some extent.  Figure 1 shows the network structure of the LSTM. The basic unit of the LSTM model is a memory block, which includes a memory cell and three gate structures that control the state of the memory cell, forget gate, input gate, and output gate. To be specific, the forget gate decides to forget the useless historical information from the memory cell state, the input gate decides the influence of the current input data on the memory cell state, and the output gate decides the output information.

Firstly, the information that needs to be eliminated from the cell is determined by the forget gate (*f*_*t*_) of the (14) as follows:

(14) $$\begin{array}{c} f_{t}=\sigma \left(b_{f}+W_{f}x_{t}+U_{f}h_{t-1}\right), \end{array}$$

where *σ* is the sigmoid activation function, which represents the amount of information retained, *x*_*t*_ is the current input vector, and *h*_*t*_ is the currently hidden layer vector. *b*_*t*_, *x*_*t*_, and *x*_*t*_ are the bias, the input weight, and the loop weight of the forget gate, respectively.

Next, the information state is updated in the cell. The external input gate (*i*_*t*_) is controlled by a sigmoid activation function of the (15) as follows:

(15) $$\begin{array}{c} g_{t}=\sigma \left(b_{g}+W_{g}x_{t}+U_{g}h_{t-1}\right). \end{array}$$

Meanwhile, the cell state (*C*_*t*_) is updated on the basis of *C*_*t*−1_ by (16) as follows:

(16) $$\begin{array}{c} C_{t}=f_{t}*C_{t-1}+g_{t}*\text{tan}h\left(b_{c}+W_{c}x_{t}+U_{c}h_{t-1}\right), \end{array}$$

where *C*_*t*_ represents the state of the memory cell at time *t*.

Finally, the information output is controlled by the output gate (*O*_*t*_) of the (18) as follows:

(17) $$\begin{array}{c} h_{t}=\left(O_{t}\right)\tanh\left(C_{t}\right), \end{array}$$

(18) $$\begin{array}{c} O_{t}=\sigma \left(b_{o}+W_{o}x_{t}+U_{o}h_{t-1}\right). \end{array}$$

We firstly build two models, unidirectional dual-layer LSTM model and bidirectional LSTM model, which aim to predict the average daily prices of Bitcoin after 1 day based on the prices in the previous 8 days. We compare the forecast results for different size time windows for the prices of gold/Bitcoin, respectively.

Furthermore, in order to address the problem of prediction lag, we adopt further optimizations to alleviate it and improve the accuracy of ups and downs. We expand the range of predictions, the average price 3 days after is based on the previous *n*-day prediction, and the up- and downtrend of prices (trend) is expressed in terms of yields over the next three days.

The up- and downtrend of prices (trend) is calculated by (19) as follows:

(19) $$\begin{array}{c} \text{trend}=\gamma_{1}*\frac{\text{price}\left(n+1\right)}{\text{price}\left(n\right)}+\gamma_{2}*\frac{\text{price}\left(n+2\right)}{\text{price}\left(n+1\right)}+\gamma_{3}*\frac{\text{price}\left(n+3\right)}{\text{price}\left(n+2\right)}, \end{array}$$

where *γ*_*i*_ · (*i*=1,  2,  3) is the weight of the fluctuation rate for the next three days, price(*n*) is the price of yesterday for the *n*-th day, and price(*i*) (*i* = *n* + 1, *n* + 2, *n* + 3) is the predicted price for the next three days.

The final predicted result (Result) is calculated by (20) as follows:

(20) $$\begin{array}{c} \text{Result}=\text{trend}\times \text{price}\left(n\right). \end{array}$$

We select 5 indicators as evaluation criteria for model performance: mean square error (MSE), root-mean-square deviation (RMSD), coefficient of determination (*R*^2^), mean absolute percentage error (MAPE), and accuracy of ups and downs predictions (Accuracy).

The mean square error (MSE) is calculated by (21) as follows:

(21) $$\begin{array}{c} \text{MSE}=\frac{1}{n}\sum_{i=1}^{n}{\left(y_{i}-\hat{y_{i}}\right)}^{2}, \end{array}$$

where *y*_*i*_ is the true value and $\hat{y_{i}}$ is the predicted value of *y*_*i*_.

The root-mean-square error (RMSE) is calculated by (22) as follows:

(22) $$\begin{array}{c} \text{RMSE}=\sqrt{\text{MSE}}=\sqrt{\frac{\sum_{i=1}^{n}{\left(\hat{y_{i}}-y_{i}\right)}^{2}}{n}}, \end{array}$$

where MSE is the mean square error.

The coefficient of determination (*R*^2^) is calculated by (23) as follows:

(23) $$\begin{array}{c} R^{2}=1-\frac{SS_{\text{res}}}{SS_{\text{tot}}}=1-\frac{\sum_{i=1}^{n}{\left(y_{i}-\bar{y}\right)}^{2}}{\sum_{i=1}^{n}{\left(y_{i}-\hat{y_{i}}\right)}^{2}}, \end{array}$$

where *SS*_res_ is the sum of squares of residuals, *SS*_tot_ is the total sum of squares, *y*_*i*_ is the true value, $\bar{y}$ is the mean of true values, and $\hat{y_{i}}$ is the predicted value of *y*_*i*_.

The mean absolute percentage error (MAPE) is calculated by (24) as follows:

(24) $$\begin{array}{c} \text{Mape}=\frac{100\%}{n}\sum_{i=1}^{n}\left|\frac{y_{i}-\hat{y_{i}}}{y_{i}}\right|, \end{array}$$

where *y*_*i*_ is the true value and $\hat{y_{i}}$ is the predicted value of *y*_*i*_.

### 2.3. Trading Strategies

#### 2.3.1. Strategy Establishment

**Figure 2:**
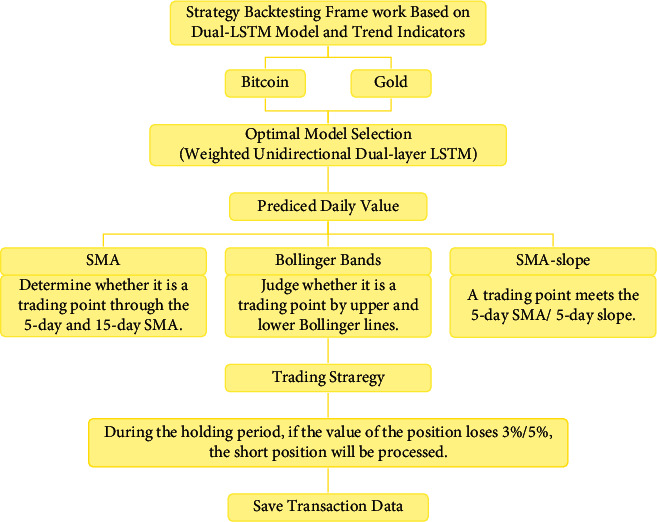
Structure diagram of the strategy backtesting framework.

**Figure 3:**
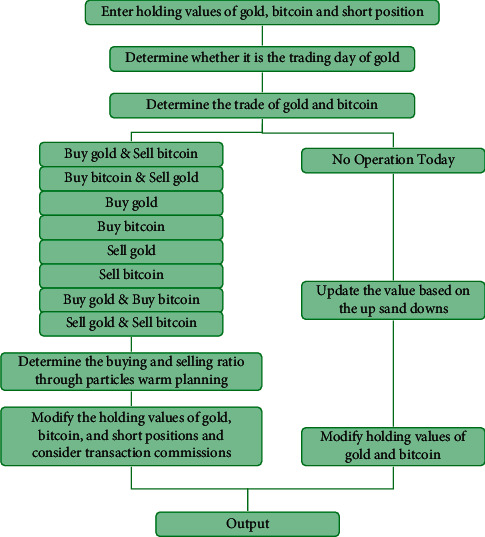
Flowchart of trading strategy.

With an initial principle of $1000, we set the specified buy position ratio, sell position ratio, and buy position ratio for the first time/after clearance before starting the trading process.  Figure 2 is the structure diagram of the strategy backtesting framework. Firstly, the daily price of gold/Bitcoin is predicted through the weighted unidirectional dual-layer LSTM model. Next, we determine whether today is a trading day and the appropriate time to buy or sell according to different strategies. In this way, we can derive nine different buy and sell combinations for gold and Bitcoin.  Figure 3 shows the detailed trading strategy. For the date of simultaneous buy or sell, we adopt the PSO to obtain the optimal buying and selling ratio of gold and Bitcoin, where the objective function is to maximize the profit. Finally, we summarize the state of the asset, including the total assets, the assets of gold, the assets of Bitcoin, and the empty asset. The experiment led by Schmidt and Traub showed that loss aversion is a common human behavior in most situations [36]. When the loss reaches 3% or 5% of the initial principle, all positions will be cleared so that the loss will be stopped in time.

#### 2.3.2. Strategy Process

*(1) Simple Moving Average Strategy*. We choose two specific indicators, the long-term simple moving average (SMA) of 15 days average daily prices and the short-term SMA of 5 days. When the short-term SMA exceeds the long-term SMA, the asset has an upward trend, implying that it is suitable for buying. When the short-term SMA moves down and intersects with the long-term SMA, the asset has a downward trend, making it suitable for selling.

*(2) Bollinger Bands Strategy*. Bollinger bands (BB) indicate areas of support and resistance. A set of parameters can be adopted according to the length of time under various situations. We select 20 days and use 2 as the multiplicative parameter before standard deviation because the proposed combination is the most commonly employed standard and interests many investors [37]. When the average daily price of gold/Bitcoin exceeds the upper Bollinger lines, the price of the asset continues to rise, which implies that a sell operation should be considered conservatively. When the average daily price of gold/Bitcoin is lower than the lower Bollinger lines, the price of the asset continues to fall, which indicates that a buying operation should be considered conservatively.

*(3) SMA-Slope Strategy*. We establish a new strategy called the SMA-slope strategy by introducing a new concept of *k*-slope based on SMA. The parameter *k* of the *k*-slope refers to the number of days where the slope is consecutively positive/negative. The *k*-slope is used to increase the buy and sell points, which promote the investment to generate more excellent interest rates.

We calculate the ratio of positive/negative slopes for gold/Bitcoin for *k* consecutive days (Table 1). It can be easily noticed that, by introducing the concept of *k*-slope, many *k* make the number of days that meet the trading conditions exceed the number of days to trade formed by SMA and BB strategies only. When *k* > 10, the number of days that meet the trading conditions is less. Therefore, we set traversals and search to range from *k* to [1, 12]. Considering that the SMA strategy has outperformed the Bollinger band strategy, we incorporate the *k*-slope into the SMA strategy to form the SMA-slope strategy, which can create more trading days than the SMA strategy. Also, it makes gold and Bitcoin more likely to be traded simultaneously, so we expect better results by using PSO.

The *k*-slope of gold/Bitcoin represents how aggressively the price rises or falls, which can be used for trend identification to establish a trading bias. A positive slope dictates a bullish bias, while a negative slope dictates a bearish bias. However, the *k*-slope follows a trend or price point, which cannot predict a trend. In order to treat the problems of possible lags, we apply the predicted price of today and the actual data of yesterday to calculate today's slope.

The *k*-slope (*s*_*i*_) is calculated by (25) as follows:

(25) $$\begin{array}{c} s_{i}=\frac{{\hat{y}}_{i}-y_{i-1}}{1\text{ day}}, \end{array}$$

where *y*_*i*−1_ is the true value on the (*i* − 1)-th day and $\hat{y_{i}}$ is the predicted value of *y*_*i*_.

When *k* > 2, if the slopes for consecutive *k* days are both positive and negative and the absolute value of the slope for the most recent day is less than the absolute value of the slope today, we will consider it as a possible buying/selling point. Directional movement is also important for analyzing the slope. When the *k-*slope continues to be positive and has a slowing trend, we decide that this is an appropriate selling point; when the *k-*slope continues to be negative and has a slowing trend, we decide that this is an appropriate buying point. To sum up, when *k* > 1, we consider *s*_*i*_ > 0 as a selling point and *s*_*i*_ < 0 as a buying point.

We set the number of consecutive days that Bitcoin has a positive slope as BITCOIN_*k*_positive, and the value that meets the requirements represents a suitable time to sell Bitcoin on that day. We set the number of consecutive days that Bitcoin has a negative slope as BITCOIN_*k*_negative, and the value that meets the requirements represents a suitable time to buy Bitcoin on that day. GOLD_*k*_positive and GOLD_*k*_negative are defined similarly. We perform a traversal search with the range from 1 to 12 for these four parameters to find the optimal value for *k* in the *k*-slope.

## 3. Results

### 3.1. Performance of LSTM Model Based on Feature Selection

**Figure 4:**
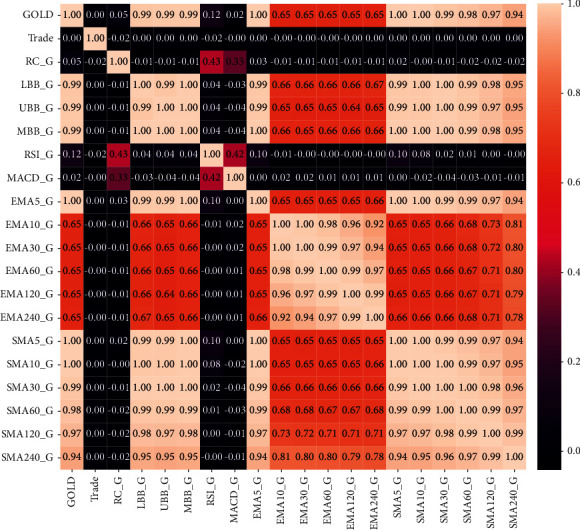
Correlation coefficients of all features with gold values.

**Figure 5:**
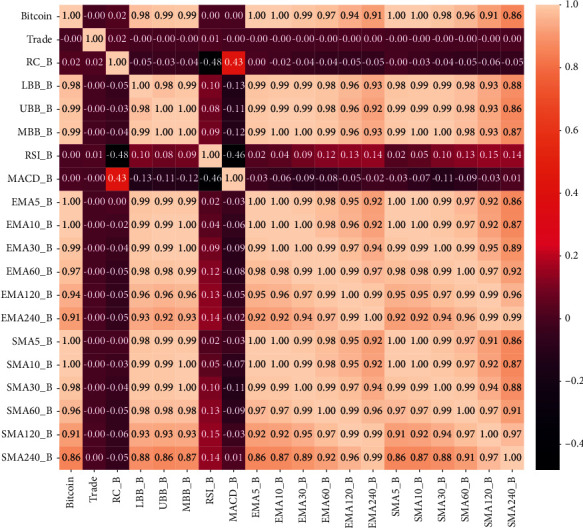
Correlation coefficients of all features with Bitcoin values.

Figures 4 and 5 represent correlation coefficients of all features with daily average price of gold/Bitcoin. The darker the colour, the smaller the influence of the feature on the value of gold/Bitcoin. Also, the lighter the colour, the greater the influence of the feature on the value of gold/Bitcoin. The value of gold has a large correlation coefficient with all features of SMA and Bollinger bands, implying a strong positive correlation. In particular, the correlation coefficients of 5-day SMA and 5-day EMA reach 1.0, which is a perfect positive correlation. Therefore, these characteristics can have a greater impact on the value of gold. The correlation coefficients of RC, RSI, and MACD were 0.05, 0.12, and 0.02, respectively, showing a positive weak correlation.

The value of Bitcoin has a strong correlation coefficient with all the characteristics of the simple moving average, exponential moving average, and Bollinger bands, even reaching 1.0 on the 5-day and 10-day SMA and EMA. Therefore, these characteristics can have a noticeable impact on the value of Bitcoin. The correlation coefficients of RC, RSI, and MACD are 0.02, 0.00, and 0.00, respectively, and there is almost no correlation. However, since only 18 indicators are selected in this paper, all indicators with weak correlations are reserved.

Table 2 represents the result of the performance comparison of LSTM models. It can be seen that the unidirectional dual-layer LSTM model is better than the bidirectional LSTM model in every index. As a result, we initially choose the unidirectional dual-layer LSTM as the basic model.

Δ*t* and *m* in Table 2 refer to the average daily price data of consecutive trading Δ*t* days as a time window to input the model training, which aims to predict the profit situation after *m* days. Therefore, it can use the predicted price data of *m* days to predict the short-term price trend.

Secondly, we compare different sizes of the time window (Δ*t*), where 1, 5, and 8 are chosen.

Table 3 represents the performance of unidirectional dual-layer LSTM model with time windows of 1, 5, and 8. It is obvious that the 5 indicators are not particularly different. Given that we pay more attention to the accuracy of ups and downs predictions (Accuracy), we choose the unidirectional dual-layer LSTM model with time windows of 5 to predict the average daily price of Bitcoin.

In the same way, a unidirectional dual-layer LSTM model with time windows of 8 is chosen to predict the average daily price of Bitcoin.

**Figure 6:**
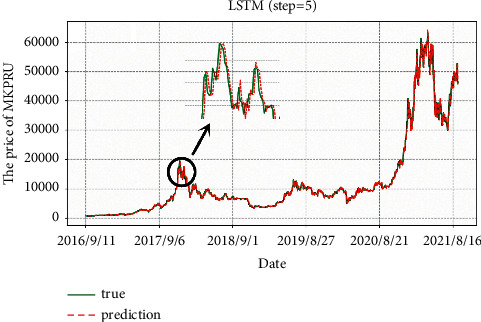
Predicted values of Bitcoin by unidirectional dual-layer LSTM model.

As shown in Figure 6, prediction lag is sometimes encountered, where the predicted average daily price of Bitcoin lags behind the change in the actual situation. It may lead to a decrease in the accuracy of ups and downs (Accuracy).

As shown in Tables 4 and 5, it can be inferred that, compared with *m* = 1, the accuracies of ups and downs (Accuracy) have more than 25% growth. Therefore, we can conclude that the model has been significantly improved. In addition, whether it is Bitcoin or gold, the result with a weight ratio of 0.4 : 0.5 : 0.1 performs better than the result with a weight ratio of 0.4 : 0.32 : 0.28. Hence, we choose a weight ratio of 0.4 : 0.5 : 0.1 to build the model.

**Figure 7:**
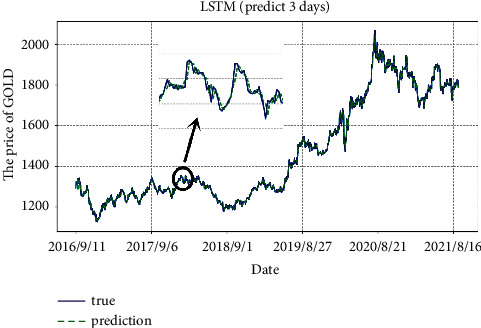
Predicted values of gold by weighted unidirectional dual-layer LSTM model.

**Figure 8:**
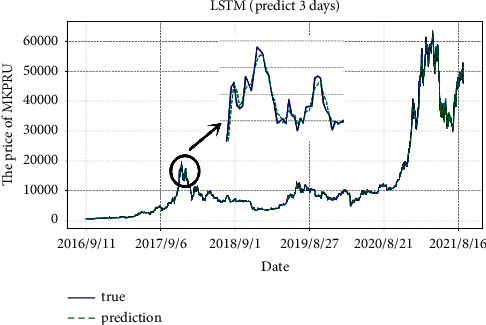
Predicted values of Bitcoin by weighted unidirectional dual-layer LSTM model.

According to the model we selected and improved, we predict the average daily prices of gold and Bitcoin, as shown in Figures 7 and 8. It can be clearly seen that the prediction lag with *m* = 3 has been alleviated. To sum up, with a time window of 5, the unidirectional dual-layer LSTM model predicting the next 3 days is the best for Bitcoin average daily price prediction. Also, with a time window of 8, the unidirectional dual-layer LSTM model predicting the next 3 days is the best one for gold average daily price prediction.

### 3.2. Financial Strategy Results

A 5-year trading simulation is conducted based on a $1000 principal. For the sake of concise and convenient representation, the symbols we will use frequently in the next two sections are explained in Table 6. The initial conditions of the simulation are as follows: the sell position ratio is 20%, the buy position ratio is 50%, the buy position ratio for the first time/after clearance is 70%, and the transaction commissions of Bitcoin and gold are 2% and 1%, respectively.

#### 3.2.1. Performance of the SMA-Slope Strategy

*(1) Optimal Value of k in k-Slope for Gold*. As the number of consecutive days with negative slopes for gold (GOLD_*k*_negative) increases, the number of buying points for gold decreases, but the upper limit of the gold asset increases. As the number of consecutive days with positive slopes for gold (GOLD_*k*_positive) increases, the number of selling points for gold decreases, but the upper limit of the gold asset increases. From Table 7, it can be concluded that long-term holding is more suitable for gold, while frequent reading operations are not appropriate. By analyzing gold assets under different settings of four parameters, we find that when GOLD_*k*_negative and GOLD_*k*_positive take 9 at the same time, the gold assets can reach the highest. Moreover, selling points have a more significant impact on gold assets because GOLD_*k*_positive can distinguish different gold assets while GOLD_*k*_negative cannot. When the buying and selling points are the same, the results of the SMA-slope strategy are better than those of the SMA strategy due to the influence of Bitcoin on gold. It increases the number of days to buy and sell both gold and Bitcoin at the same time, which contributes to better results of asset allocation.

*(2) Optimal Value of k in k-Slope for Bitcoin*. As the number of consecutive days with negative slopes for Bitcoin (BITCOIN_*k*_negative) decreases, the number of buying points for Bitcoin increases, and the upper limit of Bitcoin assets increases. As the number of consecutive days with positive slopes for Bitcoin (BITCOIN_*k*_positive) decreases, the number of selling points for Bitcoin increases, and the upper limit of Bitcoin assets increases. From Table 8, we can draw the conclusion that Bitcoin is more suitable for short-term holding. The buying point has a greater impact on Bitcoin because buying points can partition the Bitcoin asset, while selling points cannot. When the selling points are the same, the SMA-slope strategy far outperforms the SMA results because the SMA-slope strategy increases the buying point for Bitcoin. With more days to buy and sell both gold and Bitcoin simultaneously, more proper asset allocation can be obtained.

*(3) Optimal Values of k in k-Slope for the Portfolio*. We perform a global search for four *k*-slope parameters, and the optimal solution set is shown in (26) as follows:

(26) $$\begin{array}{c} \left\{\begin{array}{c} \text{BITCOIN}\_k\_\text{negative}=1,\\ \text{BITCOIN}\_k\_\text{positive}\geq 8,\\ \text{GOLD}\_k\_\text{negative}\geq 7,\\ \text{GOLD}\_k\_\text{positive}=5, \end{array}\right) \end{array}$$

Bitcoin has 1567 buying points and 52 selling points, while gold has 31 buying points and 99 selling points. This optimal solution set confirms previous conclusions; that is, selling points play a decisive role in gold assets, and buying point is crucial for Bitcoin.

However, at this time, 99% or more of the total assets are composed of Bitcoin assets, and the holding ratio also exceeds 99% of the total positions. This is a very aggressive, risky behavior that does not conform to normal human behaviors. However, this set can be ignored by setting the empty, gold, and Bitcoin ratio not to be extreme.  Table 9 shows the optimal asset results by global search.

**Figure 9:**
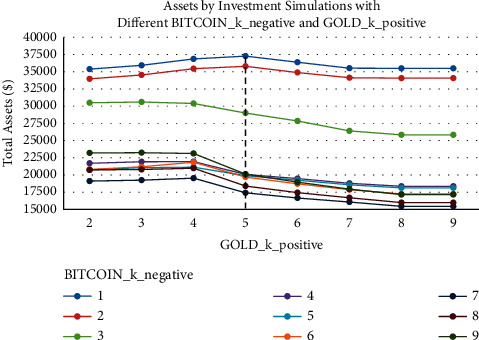
Assets by investment simulations with different BITCOIN_*k*_negative and GOLD_*k*_positive.

**Figure 10:**
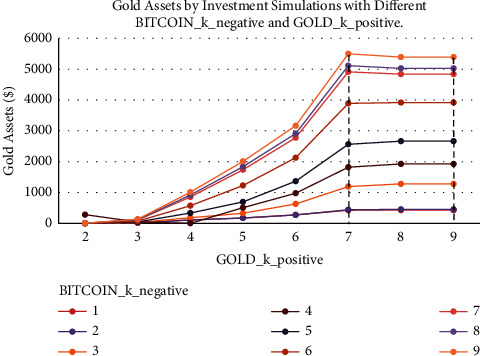
Gold assets by investment simulations with different BITCOIN_*k*_negative and GOLD_*k*_positive.

Figure 9 presents the results of different BITCOIN_*k*_negative and GOLD_*k*_positive investment simulations. It can be noticed that the best results are obtained when the value of BITCOIN_*k*_positive is 1. Similarly, Figure 10 presents the changes in gold assets at different BITCOIN_*k*_negative, and we can see that the more the proportion of Bitcoin, the smaller the proportion of gold and the more the total assets. Under different BITCOIN_*k*_negative, better results are obtained when the value of GOLD_*k*_positive is 7, but as the number of buying points for Bitcoin decreases, GOLD_*k*_positive moves to a smaller value to obtain the optimal solution. The portfolio has the tendency to decrease the proportion of gold and increase the proportion of Bitcoin in order to get a higher profit.

#### 3.2.2. Comparison of SMA, BB, and SMA-Slope Strategies

From Table 10, as for the performance of the annual percentage rate (APR) of the portfolio consisting of gold and Bitcoin, the SMA-slope strategy is better than the SMA strategy, and the SMA strategy is better than the BB strategy. The empty, gold, and Bitcoin have unbalanced asset allocations with high risk when SMA-slope and BB strategies perform best. It also confirms the possibility that investors may substitute Bitcoin for gold in a portfolio for higher risk-adjusted returns [38]. In the SMA-slope strategy, without pursuing the minimum value, we can get a set of slope values, which contributes to more balanced asset allocation, and the APR is higher than that of the SMA strategy. As shown in Table 11, the SMA-slope strategy dramatically increases the number of buying points of Bitcoin and changes the selling points of gold. It can be speculated that the SMA-slope strategy adjusts the number of trading times for gold and Bitcoin on the same day to get better results.

### 3.3. Analysis of Operational Factors Affecting Investment Results

The proper asset allocation is key to achieving the goal of high return and risk tolerance. Rebalancing will be employed when the asset allocation is out of line, which will provide the best relative return on risk [39]. Therefore, we set the BITCOIN_*k*_negative as 5 and GOLD_*k*_positive as 5 to further analyze the SMA-slope strategy. Results with these settings avoid extreme imbalances in the ratio of gold and bitcoin.

#### 3.3.1. Buy Position Ratio Analysis

**Figure 11:**
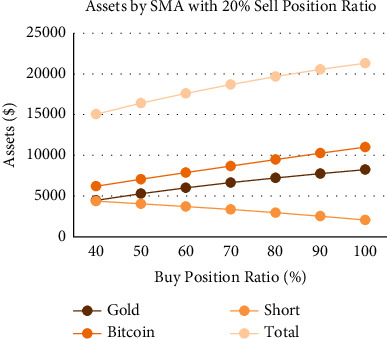
Assets by SMA strategy with 20% sell position ratio and different buy position ratio.

**Figure 12:**
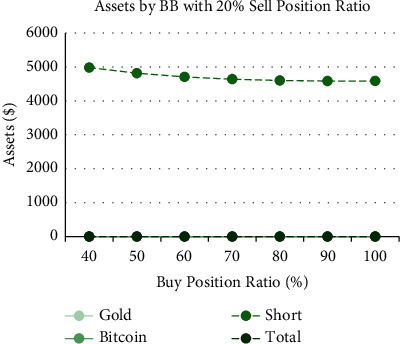
Assets by Bollinger bands strategy with 20% sell position ratio and different buy position ratio.

**Figure 13:**
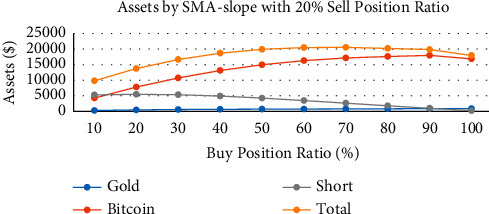
Assets by SMA-slope strategy with 20% sell position ratio and different buy position ratio.

Figures 11–13 are the analysis of buy position ratio under 3 strategies. For the SMA strategy, when we fix the gold/bitcoin sell position ratio of 20% each time and adjust the proportion of the short position each time we buy, it is evident that the APR is adjusted from 72.0% to 84.4%. The growth rate of the APR is proportional to the buy position ratio. The Bollinger band strategy shows that its annual interest rate has nothing to do with the buying position ratio, implying that the strategy is highly conservative. Its proportion of gold and Bitcoin is highly unbalanced. For the SMA-slope strategy, it can be found that the annual interest rate shows a parabolic trend, and the total assets will fall back as the position increases to warn investors. When the buy position ratio is adjusted to 70%, the maximum APR is 83.0%. However, as the buy position ratio becomes closer to 100%, the APR shows a downward trend.

Moreover, the SMA-slope strategy obtains a better solution when the buy position ratio is within the interval [40%,  80%]. When the buy position ratio is adjusted to 40%, the difference between the APRs of the SMA and SMA-slope strategies is at most 8%. Considering risk factors, it is generally not recommended for investors to use all amounts of cash to buy assets at one time, especially for Bitcoin, which is highly volatile. Therefore, with careful consideration, the SMA-slope strategy performs better in response to adjusting the buy position ratio.

#### 3.3.2. Sell Position Ratio Analysis

**Figure 14:**
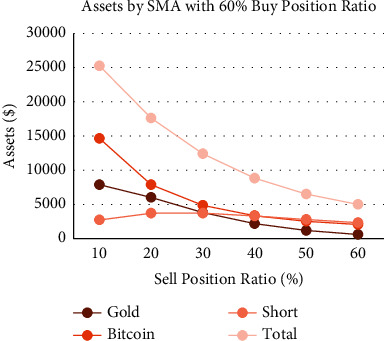
Assets by SMA strategy with 60% buy position ratio and different sell position ratio.

**Figure 15:**
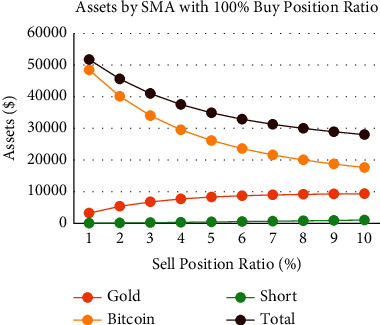
Assets by SMA strategy with 100% buy position ratio and different sell position ratio.

We try to explore the performance of the SMA strategy in extreme cases, that is, sell only a tiny percentage of gold/Bitcoin at one time. When we set the buy position ratio to 60% (Figure 14), it can be seen that the growth of the annual interest rate has a linear relationship with the sell position ratio. The higher the sell position ratio, the lower the annual interest rate. The position ratio between the holding value of Bitcoin and gold and the short position value is about 3 : 6:1, a stable ratio. We want to explore whether this ratio relationship is still satisfied in extreme cases (Figure 15). We figure out that when the sell position ratio is 1%, the final short position value is only 32.6, the value of Bitcoin holdings reaches 48436.5, and the total assets reach $51711.0, where the Bitcoin share becomes 93.7%. It is dangerous behavior. Although the data shows that a lower sell position ratio can finally greatly promote the growth of total assets, it is still a dangerous behavior requiring alertness.

#### 3.3.3. Buy Position Ratio for the First Time/after Clearance Analysis

**Figure 16:**
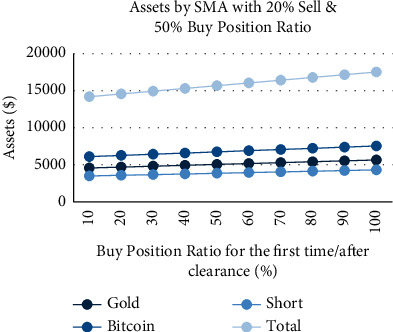
Assets by SMA strategy with 20% sell and 50% buy position ratio and different buy position ratio for the first time/after clearance.

**Figure 17:**
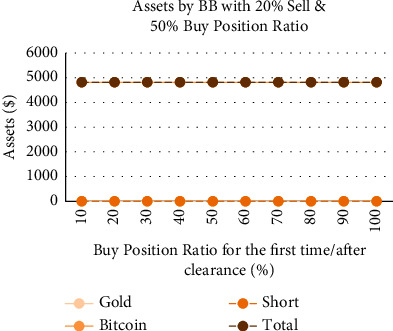
Assets by Bollinger band strategy with 20% sell and 50% buy position ratio and different buy position ratio for the first time/after clearance.

We have conducted a detailed analysis of the purchases when the current gold/Bitcoin position value is 0 under different strategies; that is, we adjust the buy position ratio for the first time/after short positions to explore its impact on the annual interest rate. We set the sell position ratio as 20% and the buy position ratio as 50%, aiming to find the changes in annual interest rate under different buy position ratios for the first time/after short positions. It can be found that, under the SMA strategy (Figure 16), the annual interest rate and the buy position ratio for the first time/after short positions have a linearly increasing relationship. Moreover, the value of empty, Bitcoin, and gold has increased, and the proportion is relatively balanced. Under the Bollinger band strategy (Figure 17), the annual interest rate has no effect on buy position ratios for the first time/after short positions, and the total assets remain at $4815.8. It confirms the conservative nature of the Bollinger band strategy again, avoiding short positions to the greatest extent.

#### 3.3.4. Different Transaction Commissions of Gold/Bitcoin

**Figure 18:**
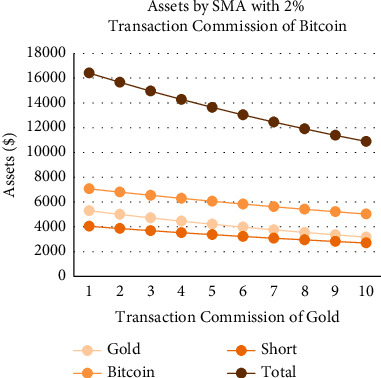
Assets by SMA strategy with 2% transaction commission of Bitcoin and different transaction commissions of gold.

**Figure 19:**
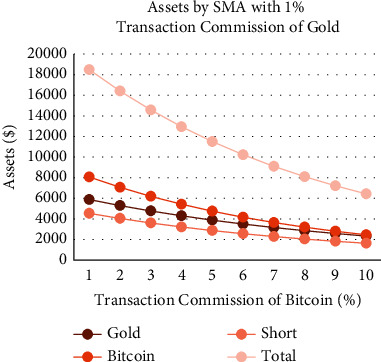
Assets by SMA strategy with 1% transaction commissions of gold and different transaction commission of Bitcoin.

**Figure 20:**
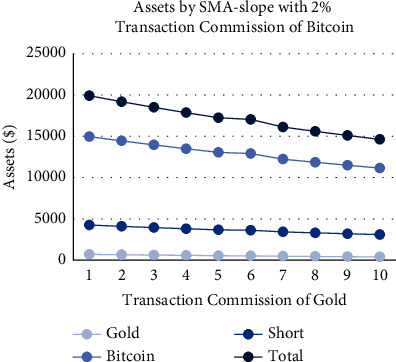
Assets by SMA-slope strategy with 2% transaction commission of Bitcoin and different transaction commissions of gold.

**Figure 21:**
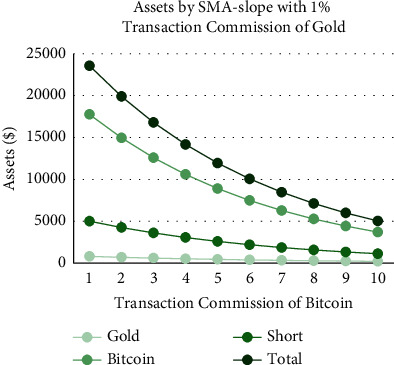
Assets by SMA-slope strategy with 1% transaction commission of gold and different transaction commissions of Bitcoin.

As shown in Figures 18–21, the assets show a linear relationship with different transaction commissions of gold/Bitcoin for SMA and SMA-slope models. Also, with the increase of the transaction commission of gold/Bitcoin, the total asset decreases linearly.

Tables 12 and 13 show the effects on the two models when the transaction commission of gold/Bitcoin changes. The average sensitivity (average rate of change) of the SMA strategy to changes in Bitcoin transaction commission is 36%, and the average sensitivity of the SMA-slope strategy is 47. Additionally, SMA and SMA-slope strategies are sensitive to gold transaction commission changes with an average sensitivity of 17%.

To sum up, due to the relatively gentle change of gold, the two models are less sensitive to changes in the transaction commission. However, because of the significant volatility and extensive fluctuation range of Bitcoin, the two models are more sensitive to it. Moreover, the sensitivity of the SMA-slope strategy is higher than that of the SMA strategy, and the highest rate of change is 75%.

## 4. Discussion

We have built a portfolio optimization model for gold and Bitcoin with high profitability to help investors make trading decisions. By sliding window and a weighted method for three days, we achieve a high accuracy of ups and downs for the unidirectional dual-layer LSTM model. Also, with the introduction of the *k*-slope and slight adjustments for the asset allocation, higher returns are realized by the SMA-slope strategy compared to common SMA and BB strategies. However, there are still some improvements that can be completed in the future work. Firstly, risk factors should be taken into more consideration. The current optimal set is a hazardous solution with highly unbalanced asset allocation. A fixed rule and some rebalancing methods can be conducted to avoid risks caused by inappropriate portions of different assets. Secondly, the relationship between gold and Bitcoin can be explored while we currently train and test the LSTM model for prediction separately. The internal influence mechanism between gold and Bitcoin may play an essential role in predicting daily prices [40]. Thirdly, the window size can be dynamic as time goes by. Fix window size is used in the current model, limiting temporal modeling in deep learning neural networks because the data defined by the window size is only modeled and unsuitable for dealing with long-term dependencies in time-series data [41]. Also, the sliding window may be more suitable for Bitcoin than gold. Finally, in the context of Industry 4.0, AI technology can be applied more in the prediction model and the simulation for practical trading processes. Many alternatives for the LSTM model may contribute to higher accuracy for daily price prediction. AI can be described as a set of techniques for modeling and simulation environmental systems, such as artistic neural networks and reinforcement learning [42]. More use of AI technology may make trading simulations more realistic.

## 5. Conclusion

This paper establishes a portfolio optimization model for gold and Bitcoin, including a weighted unidirectional dual-layer LSTM model to predict the daily prices and the SMA-slope strategy for trading decision-making. We also carry out trading simulations for gold and Bitcoin with an initial principal of $1000 for further exploration. The weighted unidirectional dual-layer LSTM model is trained by daily prices of gold/Bitcoin and their related financial indicators. In order to make the simulation more in line with the actual, we adopt a sliding window for daily price prediction. For evaluation matrices, in addition to some common indicators like *R*^2^ and RMSE, we pay more attention to the accuracy of ups and downs, which has a more direct relationship with trading decision-making. Changes in the financial market do not always obey the regular common rules or follow the same cycle. The predicted prices are obtained for the following 3 days, and a weighted method is used on them for higher accuracy of ups and downs, which reduces prediction lags to catch trends of prices better. In this paper, we compared three strategies, SMA strategy, BB strategy, and SMA-slope strategy. By analyzing results from various simulations, the SMA-slope strategy is considered the best choice for obtaining relatively high returns and avoiding extremely unbalanced asset allocation, where four parameters in the *k*-slope can be adjusted to achieve different outcomes. We focus on the operation derails involved in the investment processes and conclude that buy position ratios, sell position ratios, buy position ratios for the first time/after clearance, and transaction commissions of gold/Bitcoin all significantly impact the assets, which also have a more substantial effect on the performance of strategies.

## Figures and Tables

**Table 1:** The ratio positive/negative slopes for gold/Bitcoin for *k* consecutive days.

*k*	Gold (1826 days)									Bitcoin (1826 days)								
	2	3	4	5	6	7	8	9	10	2	3	4	5	6	7	8	9	10
Positive (%)	38	32	28	26	24	22	21	20	19	42	36	32	30	27	25	24	23	23
Negative (%)	34	27	24	21	19	18	16	15	14	31	25	21	19	16	15	13	12	11

**Table 2:** Performance comparison of LSTM models.

Model	Δ*t*	*m*	MSE	RMSE	*R* ^2^	MAPE	Accuracy (%)
Unidirectional dual-layer LSTM	8	1	751150.9776	866.6897	0.9962	2.8881	46.9163
Bidirectional LSTM	8	1	752803.4835	867.6425	0.9962	2.8641	45.5044

**Table 3:** Performance comparison with different sizes of the time window of the LSTM model.

Model	Δ*t*	*m*	MSE	RMSE	*R* ^2^	MAPE	Accuracy (%)
Unidirectional dual-layer LSTM	1	1	731372.9356	855.2034	0.9963	2.9109	46.6410
	5	1	739981.2836	860.2216	0.9962	2.8791	47.7974
	8	1	751150.9776	866.6897	0.9962	2.8881	46.9163

**Table 4:** Performance comparison with different weights for gold of the LSTM model.

Model	*γ*_1_	*γ*_2_	*γ*_3_	MSE	RMSE	*R* ^2^	Mape	Accuracy (%)
Unidirectional dual-layer LSTM(Δ*t*=8, *m*=3)	0.4	0.32	0.28	354788.3113	595.6410	0.9982	2.1194	73.7885%
	0.4	0.5	0.1	253736.0019	503.7221	0.9987	1.5871	82.0485%

**Table 5:** Performance comparison with different weights for Bitcoin of the LSTM model.

Model	*γ*_1_	*γ*_2_	*γ*_3_	MSE	RMSE	*R* ^2^	Mape	Accuracy (%)
Unidirectional dual-layer LSTM(Δ*t*=5, *m*=3)	0.4	0.32	0.28	354788.3113	595.6410	0.9982	2.1194	73.7885
	0.4	0.5	0.1	253736.0019	503.7221	0.9987	1.5871	82.0485

**Table 6:** Notations used in this section.

Symbol	Definition
Gold	Accumulated holding value of gold.
Bitcoin	Accumulated holding of Bitcoin.
Short	Cumulative remaining value of assets.
Total	Cumulative total value of assets.
Buy position ratio	The ratio of value of assets that are bought to the cumulative remaining value of assets.
Sell position ratio	The ratio of value of assets that are sold to the cumulative remaining value of assets.
Buy position ratio for the first time/after clearance	Buy position ratio for first purchase at the beginning and the first purchase after short position.

**Table 7:** Number of buying and selling points for gold and corresponding asset values at different slopes.

GOLD_k_negative	Gold buying points	Gold assets range ($)	GOLD_k_positive	Gold selling points	Gold assets range ($)
10,11,12	31	[2.58, 5473.44]	10,11,12	37	[863.20, 5304.65]
**9**	**31**	**[2.61, 5497.88]**	**9**	**40**	**[894.96, 5497.88]**
7,8	31	[2.58, 5473.44]	8	40	[894.96, 5371.01]
6	33	[1.92, 3979.27]	7	45	[905.68, 5473.44]
5	36	[1.78, 3664.29]	6	67	[553.25, 3264.70]
4	45	[1.32, 1487.55]	5	99	[349.40, 1990.88]
3	63	[1.34, 1042.88]	4	156	[173.06, 1056.99]
2	98	[1.20, 960.06]	3	298	[33.91, 138.46]
			2	576	[1.20, 4.04]
SMA	31	[2.61, 5473.44]	SMA	37	[877.41, 5181.83]

**Table 8:** Number of buying points and selling points for Bitcoin and corresponding asset values at different slopes.

BITCOIN_k_negative	Bitcoin buying points	Bitcoin assets range ($)	BITCOIN_k_positive	Bitcoin selling points	Bitcoin assets range ($)
11,12	46	[1117.15, 6960.06]	**8,9,10,11,12**	**52**	**[1127.11, 34864.58]**
10	47	[1102.19, 7181.45]	7	54	[1111.62, 33071.92]
9	48	[1118.53, 6994.15]	6	57	[1093.92, 31634.04]
8	51	[970.53, 6676.25]	5	62	[1081.70, 30123.10]
7	54	[1026.62, 6351.62]	4	73	[1064.18, 27314.72]
6	70	[1565.28, 8649.57]	3	103	[1068.01, 23883.65]
5	108	[2062.81, 11227.64]	2	155	[1026.62, 20596.88]
4	175	[2434.13, 12411.84]	1	239	[970.53, 18015.70]
3	336	[4101.21, 21453.82]			
2	735	[6283.13, 32608.38]			
**1**	**1567**	**[7816.49, 34864.58]**			
SMA	46	[1226.95, 7127.42]	SMA	52	[1226.95, 7127.42]

**Table 9:** Optimal asset results by global search.

BITCOIN_k_negative	BITCOIN_k_positive	GOLD**_k_negative**	GOLD**_k_positive**	Empty ($)	Gold assets ($)	Bitcoin assets ($)	Total assets ($)
1	8	7	5	134.641	176.012	36950.113	37260.767
1	8	8	5	134.659	176.035	36954.796	37265.490
1	8	9	5	134.659	176.035	36954.796	37265.490
1	9	7	5	134.659	176.035	36954.796	37265.490
1	9	8	5	134.659	176.035	36954.796	37265.490
1	9	9	5	134.659	176.035	36954.796	37265.490
1	Null	7	5	134.659	176.035	36954.796	37265.490
1	8	Null	5	134.659	176.035	36954.796	37265.490
1	Null	Null	5	134.659	176.035	36954.796	37265.490

**Table 10:** Comparison of the results of different strategies.

Strategy					Empty ($)	Gold assets ($)	Bitcoin assets ($)	Assets ($)	Annual percentage rate (APR) (%)
BITCOIN_k_negative	BITCOIN_k_positive	GOLD_k_negative	GOLD_k_positive						
**SMA-slope**	**1**	**8**	**7**	**5**	**134.64**	**176.01**	**36950.11**	**37260.77**	**106.18**
SMA-slope	1	Null	Null	9	159.22	426.08	34906.09	35491.40	104.19
SMA-slope	5	Null	Null	5	4251.05	696.73	14953.83	19901.61	81.88
SMA-slope	5	NULL	Null	9	3653.54	2664.75	11812.30	18130.59	78.52
SMA-slope	9	Null	Null	7	4411.51	5497.70	8037.65	17946.8	78.15
SMA					3976.98	5181.83	6960.06	16118.87	74.37
BB					≈0	≈0	4950.74	4950.74	37.70

**Table 11:** Selling and buying points for different strategies.

Strategy	Bitcoin buying points	Bitcoin selling points	Gold buying points	Gold selling points
BB	449	76	50	1105
SMA	46	52	31	37
SMA-slope (best)	1567	52	31	99

**Table 12:** Effects on SMA and SMA-slope strategies by changes in transaction commission of Bitcoin.

Transaction commission of gold (%)	Transaction commission of Bitcoin (%)	SMA		SMA-slope	
		Total assets ($)	Rate of change (%)	Total assets ($)	Rate of change (%)
1	1	18490.8	−13	23570.5	−18
1	2	16410.6	0	19901.6	0
1	3	14570.8	11	16794.1	16
1	4	12943	21	14163.5	29
1	5	11502.6	30	11937.9	40
1	6	10227.5	38	10056.2	49
1	7	9098.4	45	8466	57
1	8	8098.3	51	7123.1	64
1	9	7212.1	56	5989.6	70
1	10	6426.6	61	5033.5	75

**Table 13:** Effects on SMA and SMA-slope strategies by changes in transaction commission of gold.

Transaction commission of gold (%)	Transaction commission of Bitcoin (%)	SMA		SMA-slope	
		Total assets ($)	Rate of change (%)	Total assets ($)	Rate of change (%)
1	2	16410.6	−5	19901.6	−4
2	2	15663	0	19179.9	0
3	2	14953.4	5	18497.2	4
4	2	14279.6	9	17850.9	7
5	2	13639.9	13	17238.6	10
6	2	13032.3	17	17027.6	11
7	2	12455.2	20	16107.4	16
8	2	11906.9	24	15584.6	19
9	2	11385.9	27	15088	21
10	2	10890.9	30	14616	24

## Data Availability

The data included in this paper are available without any restriction.
